# Circulating Long Noncoding RNA as a Potential Target for Prostate Cancer

**DOI:** 10.3390/ijms160613322

**Published:** 2015-06-11

**Authors:** Yin-Jie Su, Jin Yu, Ya-Qin Huang, Jin Yang

**Affiliations:** 1 Trainee Brigade, the Third Military Medical University, Chongqing 400038, China; E-Mail: yinjiesu@tmmu.edu.cn; 2 Department of Cell Biology, the Third Military Medical University, Chongqing 400038, China; E-Mails: yujin100@aliyun.com (J.Y.); hyq808024@163.com (Y.-Q.H.)

**Keywords:** long noncoding RNA, prostate cancer, biomarker, therapeutic target

## Abstract

Prostate cancer is considered the second most common visceral malignancy in men in Western countries. Its emergence is largely due to the coordination of a malignant network, and long noncoding RNA has been recently demonstrated to play a critical role in prostate carcinogenesis. The aberrant expression of long noncoding RNA in prostate cancer patients is strongly associated with diagnosis, risk stratification and carcinogenesis, information that provides new insight into the complicated intracellular milieu of prostate cancer. This review focuses mainly on literature evidence for the role of long noncoding RNA in prostate cancer, which may suggest novel strategies for its prognosis, diagnosis and clinical treatment.

## 1. Introduction

Prostate cancer is considered to be the second most common visceral malignancy in men in Western countries [1,2]. According to the Cancer Statistics report in 2014, there will be an estimated 233,000 new cases, representing 27% of all cancer cases, and an estimated 29,480 deaths, representing 10% of cancer-related deaths among American men [3]. Prostate cancer is more likely to affect older people than younger people, and 60% of new cases are diagnosed in patients older than 60 years of age. Most diagnoses are made in the terminal stage due to the lack of specific and sensitive methods for early prostate cancer screening [4,5]. Although pathological biopsy is considered the gold standard for cancer diagnosis, the invasiveness and risk of adverse effects limit its clinical use. Hence, there is a significant need to develop a noninvasive assay.

Serum prostate specific antigen (PSA) has been widely used as the optimal conventional serum marker for prostate cancer in the clinical setting, especially as a marker for initial diagnosis, as it can predict cancer risk and treatment outcome [6]. According to the literature, approximately 240,000 individuals are diagnosed with prostate cancer in the United States annually; however, fewer than 15% eventually die, which has been largely attributed to the widespread application of PSA testing and the effective treatment of early-stage prostate cancer [1,7,8,9,10]. Although PSA testing has positively influenced the diagnosis and treatment of prostate cancer, significantly decreasing the prostate cancer-related mortality rate [7,8,9,10,11], it has drawbacks that have negatively affected certain men. For example, PSA is specific to the prostate but not to prostate cancer. PSA can be elevated in some benign situations, such as prostatic hyperplasia [12] and prostatitis [13], and PSA levels can change frequently and inconsistently depending on the patient’s condition. Additionally, serum PSA levels are not specifically correlated with malignancy. According to certain studies, the morbidity of more than a quarter of patients with localized prostate cancer is closely related to unnecessary overtreatment and the missed diagnosis of life-threatening cancer [14,15,16,17,18]. Therefore, the shortcomings of the PSA test highlight its insufficiency and risks in prostate cancer detection. Thus, the development of new and effective methods is urgently needed for clinical prostate cancer screening.

Long noncoding RNAs constitute a large proportion of noncoding transcripts that contain more than 200 nucleotides, and they have recently emerged as a new player in cancer biology [19]. Previously considered to be transcriptional noise, long noncoding RNAs have now been demonstrated to play a key role in carcinogenesis [20,21]. In cancer, the aberrant expression of long noncoding RNAs contributes to the development and progression of cancer by influencing proliferation, metastasis, self-renewal, survival and apoptosis through either transcriptional or post-transcriptional regulation. These aberrantly expressed long noncoding RNAs can be detected in circulating cancer cells and in the serum and urine of cancer patients, and they exhibit specific expression patterns at various stages of cancer and in various tissues. Thus, the evidence suggests that long noncoding RNA may serve as an effective biomarker for cancer detection [22]. In this review, we not only focus on the role of long noncoding RNAs in prostate cancer diagnosis but also discuss their biological behaviors in prostate cancer.

## 2. Long Noncoding RNAs as Potential Prognostic Markers for Detecting Prostate Cancer

### 2.1. Single Biomarker Tests

Neoplastic transcriptomes are more complex than previously believed. Along with the dysregulation of protein coding genes and small noncoding RNAs, misexpression of long noncoding RNAs has also proven to be a central contributor to carcinogenesis. Considering that long noncoding RNAs are detectable at the serum/urine level and exhibit specific expression levels at various stages of prostate cancer, selecting a long noncoding RNA as a prostate cancer biomarker would be a breakthrough in clinical prostate cancer screening. In 1999, Bussemakers and colleagues first reported *DD3^PCA3^*, a long noncoding RNA, as a potential diagnostic biomarker for prostate cancer [23]. They found that *DD3^PCA3^* is a unique, polyadenylated, atypical, alternatively spliced noncoding RNA that is extremely specific to the prostate, with amplification in 18 different normal human prostate tissues. Moreover, in a cohort of 56 patients with prostatic tumors, 53 patients over-expressed this RNA in prostatic tumor tissue compared with non-neoplastic prostatic tissue from the same patient. To further explore the promising role of *DD3^PCA3^* in prostate cancer detection, the level of *DD3^PCA3^* in the urine of patients with varying stages of prostate cancer was assessed. Researchers found that by examining the level of *DD3^PCA3^* in 201 urine samples, the overall sensitivity, specificity and positive and negative predictive values for detecting prostate cancer were 82%, 76%, 67% and 87%, respectively, compared with the values for urinary PSA of 98%, 5%, 40% and 83%, respectively [24]. Among 108 patients with a PSA level >3 ng/mL, there were only 24 individuals who had prostate cancer confirmed by a biopsy, whereas 16 of the 24 patients were positive for *DD3^PCA3^* [25]. These data indicate that *DD3^PCA3^* may be a superior biomarker to *PSA* in prostate cancer detection. First, *DD3^PCA3^* is a prostate-specific gene and is particularly over-expressed in more than 95% of prostate cancers. Second, the *DD3^PCA3^* test exhibits a higher specificity than the conventional PSA test because *DD3^PCA3^* levels do not change constantly or rely on the patient’s condition. Moreover, the *DD3^PCA3^* scores predict prostatic malignancy more accurately than PSA, which could potentially reduce the number of unnecessary biopsies, overtreatment and the rate of missed diagnoses [25,26,27,28].

Recent reports have demonstrated that a novel long noncoding RNA is also involved in prostate cancer detection. Metastasis associated lung adenocarcinoma transcript 1 (*MALAT1*) was used to predict metastasis and survival in patients with early-stage non-small cell lung cancer [29]. Ren *et al.* found that its derived mini-RNA (*MD-miniRNA*) can be used in a novel approach to detect human prostate cancer [30]. The serum *MD-miniRNA* levels were significantly elevated in a cohort of 196 patients compared with non-prostate cancer patients. At a cut-off of 867.8 copies/mL plasma, the sensitivity and specificity between prostate cancer patients and non-prostate cancer patients were 58.6% and 84.8%, respectively, and the sensitivity and specificity between positive and negative biopsies were 43.5% and 81.6%, respectively. Additionally, Wang *et al.* reported that the *MALAT1* model would prevent approximately 30.2%–46.5% of unnecessary biopsies in patients with serum PSA levels of 4–10 ng/mL [31]. Moreover, Ren *et al.* quantitatively measured the expression of *MALAT1* by real-time PCR in prospectively collected urine samples and found that *MALAT1* expression was closely associated with the Gleason score and tumor size [32]. Thus, these data indicated that *MALAT1* is a promising biomarker for prostate cancer detection.

Additionally, other long noncoding RNAs have also been identified as potential tools for the risk stratification of patients with prostate cancer. *PCAT-18* (prostate cancer-associated noncoding RNA transcript 18), a long noncoding RNA, was recently discovered by RNA sequencing; *PCAT-18* exhibits a highly specific expression pattern in prostate cancer. This gene is specifically expressed in prostate tissue and is up-regulated in prostate cancer compared with other benign and malignant tissues. Similar to the aforementioned long noncoding RNAs, *PCAT-18* can be detected in plasma, and its expression incrementally increases as prostate cancer progresses from localized to metastatic disease. These results implicate *PCAT-18* as a potential biomarker for metastatic prostate cancer [33]. A similar study was conducted by Sun *et al.* in 1997 [34], who found that *PTI-1* (prostate tumor inducing gene-1) is differentially expressed in the blood in patients with prostate cancer *versus* those with benign prostatic hypertrophy or a normal prostate and that serum PTI-1 levels can predict the tumor volume, though there was only one cancer cell present in 10^8^ *PTI-1*-negative cells. This study indicated that PTI-1 represents a sensitive marker for prostate cancer progression. Unlike other serum/urine-based biomarkers, *SChLAP1* (second chromosome locus associated with prostate-1; also called *LINC00913*), second chromosome locus associated with prostate-1, was identified as a tissue-based biomarker of clinical outcome after radical prostatectomy in patients with localized prostate cancer [35]. *SChLAP1* expression increased with prostate cancer progression, and a high level of *SChLAP1* was associated with poor outcome among patients with clinically localized prostate cancer after radical prostatectomy.

### 2.2. Multi-Biomarker Tests

Although the abovementioned long noncoding RNAs are promising predictive tools for cancer detection, their accuracy is not better than that of a biopsy. The specificity and sensitivity of *DD3^PCA3^*, *MALAT1*, and *EN2* (engrailed homeobox 2) are 82% and 76%, 58.6% and 84.8%, and 66% and 88.2%, respectively. Other long noncoding RNAs are not 100% accurate for risk stratification and do not predict the clinical outcome of prostate cancer patients. Additionally, Lee and colleagues found that *DD3^PCA3^* was not detected in all of the tested samples from patients with prostate cancer [36]. There remains a discrepancy between these biomarkers and the actual clinicopathological features of these patients, which can lead to unnecessary overtreatment and missed diagnoses of life-threatening cancer. To reduce these insufficiencies and the risks of depending on a single biomarker, multivariate prostate cancer biomarker tests should be developed. For example, the limitation of *DD3^PCA3^* as a biomarker can be improved by combining it with other molecular markers, such as the *TMPRSS2-ERG* fusion gene (the transmembrane protease, serine 2 gene and the v-ets erythroblastosis virus E26 oncogene homolog (avian) gene) [37]. The ability of the expression pattern of nine prostate-related genes to predict prostate cancer was evaluated in a cohort of 106 patients who underwent prostatectomies [38]. The quantification of the nine prostate-related genes (*AibZIP*, *D-GPCR*, *EZH2*, *PCA3*, *PDEF*, *prostein*, *PSA*, *PSCA*, and *TRPM8*) by real-time PCR and the receiver operating characteristic (ROC) analysis of their expression for risk stratification revealed that PCA3 was the best single marker with the highest area under the curve (AUC = 0.85), whereas a multivariable logistic regression model (*EZH2*, *PCA3*, *prostein*, and *TRPM8*) showed even greater predictability than PCA3 alone, with an AUC of 0.9. Moreover, Lee *et al.* identified six long noncoding RNAs (*AK024556*, *XLOC_007697*, *LOC100287482*, *XLOC_005327*, *XLOC_008559*, and *XLOC_009911*) that were significantly up-regulated in all the prostate cancer patient urine samples compared with the normal urine samples [36]. Therefore, the above data indicate the potential utility of these long noncoding RNAs in a multi-biomarker method to successfully distinguish cancerous from normal tissues.

### 2.3. Long Noncoding RNAs and Gene Variance in Prostate Cancer

Analysis of the differences in genetic variance between populations has always been considered as a method for the risk stratification of diseases. Long noncoding RNAs constitute a large portion of the transcriptome, and their genetic variance was found to be strongly correlated with the risk stratification of patients with prostate cancer.

*PCGEM1* (prostate cancer gene expression marker 1), a long noncoding RNA, has a significantly higher expression level in prostate cancer cells in African-American men [39]. A study by Xue *et al.* indicated that polymorphisms in *PCGEM1*, specifically the two tSNPs (tagged single nucleotide polymorphisms) rs6434568 and rs16834898, are associated with the risk of prostate cancer in Chinese men [40]. Additionally, polymorphisms of another long noncoding RNA, *PCA3*, were associated with the risk of prostate cancer. Zhou *et al.* found that individuals with a short tandem TAAA repeat polymorphism in the PCA3 promoter have a higher risk of prostate cancer than individuals with fewer TAAA repeats [41]. Moreover, Chinese researchers [42] revealed the complex landscape of genomic alterations in Chinese patients with prostate cancer by analyzing the transcriptomes of 14 pairs of prostate cancer and adjacent normal tissues. Two novel gene fusions, *CTAGE5-KHDRBS3* and *USP9Y-TTTY15*, were identified that occur repeatedly in Chinese patients, with frequencies of 37% and 35.2%, respectively. However, the *TMPRSS2-ERG* fusion appeared at a much lower frequency (21.4%) than it does in Caucasian patients. Taken together, these results suggest a new role for long noncoding RNAs in prostate cancer beyond transcriptional regulation, as they additionally contribute to new methods for risk prediction in patients with prostate cancer based on genetic polymorphisms.

## 3. Long Noncoding RNAs as Potential Therapeutic Targets in Prostate Cancer

Long noncoding RNAs constitute a large cluster of transcripts; besides their promising role in clinical prostate cancer screening, these RNAs play an essential role in prostate cancer biology. These RNAs may (1) mediate prostate carcinogenesis through the androgen receptor (AR); (2) act as an oncogene or a tumor suppressor gene; and (3) participate in the process of DNA repair and tumor metabolism during prostate carcinogenesis. The studies discussed below have revealed that prostate cancer cell fates can be significantly influenced by interfering with the malignant network in which long noncoding RNAs participate. Hence, it will be interesting to develop new clinical therapeutic interventions based on the mechanisms of action of long noncoding RNAs in prostate cancer.

### 3.1. Long Noncoding RNAs Mediate Prostate Carcinogenesis through the Androgen Receptor

Hyperactive AR remains a key determinant in prostate cancer carcinogenesis and resistance to current therapies. Prostate cancer cells rely uniquely on androgens for proliferation [43], and blocking the AR pathway with androgen deprivation therapy invariably induces tumor regression [44,45]. However, in later stages, almost all prostate cancer cases inevitably progress to recurrent castration-resistant prostate cancer (CRPC), becoming androgen-independent and acquiring the ability to metastasize [46]. Although the mechanisms involved in regulating AR activity are poorly understood, it is essential to define this molecular event to better comprehend prostate cancer progression. To clarify this process, Takayama *et al.* demonstrated that AR-regulated transcripts of unknown functions are transcribed from the intergenic or AS (anti-sense) region of genes in prostate cancer by employing an AR transcriptional network analysis. This evidence indicates that the AR-regulated transcripts of unknown function may include a series of noncoding RNAs [47,48]. As mentioned above, long noncoding RNAs make up a large proportion of noncoding RNAs, and their presence affects prostate cancer viability and susceptibility. Hence, it is of great interest to elucidate the regulatory mechanisms involving AR and long noncoding RNAs in prostate cancer.

*PlncRNA-1*, prostate cancer up-regulated long noncoding RNA 1, has been reported to be up-regulated in prostate cancer and to be involved in reciprocal communication with AR, which contributes to prostate carcinogenesis [49]. Silencing *PlncRNA-1* expression inhibits the proliferation but promotes the apoptosis of prostate cancer cells as well as reduces the expression of AR mRNA, AR protein and its downstream target *NKX3.1*, a highly expressed prostate tissue-specific gene that is rapidly activated in response to AR signaling [50]. Notably, blocking AR signaling also suppresses *PlncRNA-1* expression. Taken together, these results demonstrate a mutual feedback loop between *PlncRNA-1* and AR that may contribute to prostate carcinogenesis.

CTBP1-AS, a long noncoding RNA, is transcribed from the AS region of the C-terminal binding protein 1 (*CTBP1*) gene. It is predominantly localized in the nucleus and is up-regulated in prostate cancer to promote the growth of both hormone-dependent and castration-resistant cancer [51,52]. Mechanistically, *CTBP1-AS* directly represses *CTBP1* expression by recruiting the RNA-binding transcriptional repressor PSF and histone deacetylases. *CTBP1-AS* also exhibits global androgen-dependent functions by inhibiting tumor suppressor genes via the PSF-dependent mechanism, thereby promoting cell cycle progression [51].

Of the long noncoding RNAs involved in prostate carcinogenesis, *PCGEM1* and *PRNCR1* (prostate cancer noncoding RNA1) have been identified as playing critical roles via coordination with AR [53]. *PCGEM1* is a prostate tissue-specific gene encoded on chromosome 2q32 that serves as a prostate cancer gene expression marker [54]. Evidence has revealed that this gene is expressed in prostate cancer, particularly in AR-positive cell lines. *PCGEM1* over-expression promotes proliferation and colony formation [39] and inhibits doxorubicin-induced apoptosis in prostate cancer cells [55]. However, whether the resistance to cell death that is associated with this gene in prostate cancer is mediated by AR must be further elucidated. *PRNCR1*, a 13-kb long noncoding RNA that is transcribed from chromosome 8q24, is involved in maintaining prostate cancer susceptibility [56]. Its inhibition attenuates the viability of prostate cancer cells and decreases the transactivation activity of AR, which indicates that *PRNCR1* could be involved in prostate carcinogenesis through an AR-mediated pathway. However, in a large cohort of more than 230 individuals, Prensner *et al.* observed that neither *PCGEM1* nor *PRNCR1* interacted with AR, and neither are components of AR signaling [57]. These findings refute the argument that these two long noncoding RNAs interact with AR signaling.

As we mentioned before, almost all prostate cancer cells inevitably progress in later stages to recurrent CRPC, which is androgen-independent and has the ability to metastasize. This begs the question of what mechanisms regulate this transition from AR-dependence to AR-independence. Wang *et al.* found that *Linc00963* (long intergenic non-protein coding RNA 00963) may participate in this transition by affecting EGFR (epidermal growth factor receptor) activity [58]. In their study, Linc00963 was over-expressed twofold in a hormone-insensitive prostate cancer cell line (C4-2) compared with the hormone-sensitive LNCaP cell line. Inhibiting Linc00963 expression attenuated the apoptosis, migration and invasion of C4-2 cells and down-regulated EGFR and its downstream gene p-AKT (phosphorylated protein kinase B). Specifically, these results suggest that studying long noncoding RNAs and AR-mediated signaling pathways may provide a better understanding of prostate carcinogenesis.

### 3.2. Long Noncoding RNAs Mediate Prostate Carcinogenesis by Acting as Oncogenes and Tumor Suppressor Genes

In normal cells, the cooperation between oncogenes and tumor suppressor genes maintains intracellular homeostasis. However, this delicate balance is usually disrupted in cancer. In cancer, most long noncoding RNAs are over-expressed and act as oncogenes (onco-lncRNA), whereas some long noncoding RNAs are underexpressed and act as tumor suppressors (TS lncRNA). Concentrating on the roles of these onco-lncRNAs and TS lncRNAs in prostate cancer to develop effective interventions to interrupt their behavior is a potential promising way to target prostate cancer.

#### 3.2.1. Long Noncoding RNAs as Oncogenes

Inducing cell cycle arrest and activating apoptosis are common features of DNA damage response pathways that remove aberrant cells from the body. Certain long noncoding RNAs function in opposition to these mechanisms by acting as oncogenes. *PCAT-1* (prostate cancer-associated intergenic non-coding RNA transcript 1) is transcribed from chromosome 8q24, a locus that is associated with prostate cancer risk and susceptibility. *PCAT-1* is particularly up-regulated in a subset of high-grade localized and metastatic prostate cancers. This gene was found to promote prostate cancer progression [59]. Over-expression of *PCAT-1* increased cell proliferation, whereas its inhibition by siRNA (small interfering RNA) knockdown led to a reduced proliferation rate of 25%–50%. After knocking down *PCAT-1* in prostate cancer cells, gene expression profiling identified 255 up-regulated genes and 115 down-regulated genes. These up-regulated genes were strongly associated with mitosis and cell cycle control, whereas the down-regulated genes aided in stratifying patients into molecular subtypes, such as BRCA2 (breast cancer 2), CENPE (centromere associated protein E) and CENPF (centromere associated protein F). Therefore, these data indicate that *PCAT-1* acts as an oncogene and is an emerging molecular tool in the field of molecular research and therapy.

The long noncoding RNA *MALAT1* was recently reported to maintain prostate cancer tumorigenicity and progression. It is involved in pre-mRNA processing and regulates the alternative splicing of pre-mRNA by modulating the levels of serine/arginine splicing factors [60,61]. The aberrant expression of *MALAT1* affects the normal splicing of a subset of mRNAs. *MALAT1* is up-regulated in prostate cancer, and down-regulating it via siRNA attenuated prostate cancer cell growth, invasion and migration and induced CRPC cell cycle arrest in G0/G1 followed by concomitant prolongation of survival of tumor-bearing mice [32]. However, it remains unknown whether *MALAT1* affects normal mRNA splicing in prostate cancer.

*SChLAP1*, second chromosome locus associated with prostate-1, is a long noncoding RNA that is frequently over-expressed in aggressive prostate cancers. Prensner *et al.* found that this gene can drive malignancy by antagonizing the tumor-suppressive functions of the SWI/SNF (SWItch/Sucrose NonFermentable) complex [62]. *SChLAP1* can directly bind to SNF5 (Sucrose NonFermentable 5), a core component of the SWI/SNF complex, and prevent the SWI/SNF complex from binding to its target promoters, resulting in decreased target gene expression.

Another long noncoding RNA, *ANRIL* (antisense noncoding RNA in the INK4 locus), was also found to be critical for prostate cancer pathogenesis. This long noncoding RNA is encoded in the *INK4b-ARF-INK4a* gene cluster on chromosome 9p21.3. Elevated expression of *ANRIL* contributes to the development of prostate cancer by inhibiting a tumor suppressor gene. *ANRIL* (1) silences INK4b/ARF/INK4a (a tumor suppressor locus in normal and cancerous cells) by binding to CBX7 (chromobox homolog 7), thereby impacting the ability of CBX7 to repress the function of INK4b/ARF/INK4a and control cell senescence [63] and (2) inactivates p15(INK4B) (a tumor suppressor) by binding to SUZ12 (suppressor of zeste 12 homolog), a subunit of polycomb repressive complex 2, promotes SUZ12 binding to the p15(INK4B) locus, and represses the expression of the p15(INK4B) locus [64]. Taken together, these findings support an important role for long noncoding RNAs as oncogenes in prostate cancer and suggest that the cancer-specific functions of these long noncoding RNAs may be harnessed to “control” tumorigenesis.

#### 3.2.2. Long Noncoding RNAs as Tumor Suppressors

Similar to most tumor suppressor genes, TS lncRNAs are expressed to protect the cell from becoming cancerous. H19 is a long noncoding RNA that is transcribed from the H19/Igf2 gene cluster on chromosome 11p11.5. Evidence suggests that this long noncoding RNA plays a critical role in tumor progression by exhibiting oncogenic activity in choriocarcinoma [65] and bladder cancer [66]. However, Zhu *et al.* found that *H19* plays a tumor-suppressive role in metastatic prostate cancer by repressing the effects of TGFβ1, which is involved in metastasis [67]. *H19* and its derivative *miR-675* were both significantly down-regulated in metastatic prostate cancer cells but not in non-metastatic prostate cancer cells. Up-regulating *H19* increased *miR-675* levels and repressed cell migration, but the functions of both *H19* and *miR-675* were attenuated by TGFβ1. Moreover, *miR-675* repressed the translation of TGFβ1 by directly binding to the 3′UTR of TGFβ1. Altogether, these results indicate a suppressor role for the *H19/miR-675* axis in prostate cancer metastasis.

*GAS5*, growth arrest-specific 5, was demonstrated to promote the apoptosis of prostate cancer cells [68]. This gene is encoded at the prostate cancer-associated locus on chromosome 1q25, and its expression is decreased in CRPC cells. The over-expression of *GAS5* in prostate cancer cells reduces drug/UV resistance and promotes apoptosis. Based on the evidence stated above, long noncoding RNAs can function as transcriptional regulators, tumor suppressor genes or oncogenes in prostate carcinogenesis. Hence, the reintroduction/reactivation of TS lncRNAs and the suppression of onco-lncRNAs could provide new approaches for targeting prostate cancer. However, few long noncoding RNAs and their mechanisms of action have been elucidated. Therefore, further studies are needed to identify additional long noncoding RNAs and their mechanisms of action related to carcinogenesis so that we can better understand their regulatory networks and develop novel approaches for prostate cancer intervention.

### 3.3. Long Noncoding RNAs Mediate Prostate Carcinogenesis through Other Processes

#### 3.3.1. Long Noncoding RNAs and DNA Repair

Double-strand break (DSB) repair is a method of DNA repair, and mutations in genes involved in this process are common in many cancers. Prensner *et al.* first described the involvement of long noncoding RNAs in the mechanisms by which DSB repair is impaired [69]. These authors found that the prostate cancer outlier lncRNA *PCAT-1* can regulate the cellular response to genotoxins by repressing the expression of BRCA2 to create a deficiency in homologous recombination (HR). This evidence suggests a new mechanism for HR impairment in prostate cancer.

#### 3.3.2. Long Noncoding RNAs and Tumor Metabolism

As mentioned above, the tumorigenic activity of *PCGEM1* is not correlated with AR. Hung *et al.* reported a novel function in which *PCGEM1* promotes prostate cancer growth predominantly by regulating tumor metabolism, regardless of hormone or AR status [70]. In their study, *PCGEM1* regulated metabolism at the transcriptional level. *PCGEM1* can directly bind to c-Myc, promote the recruitment of c-Myc to chromatin, enhance the transactivation of c-Myc and ultimately affect multiple metabolic pathways, including the pentose phosphate pathway, tricarboxylic acid cycle, glucose and glutamine metabolism, and nucleotide and fatty acid biosynthesis. This study indicated that long noncoding RNAs could transcriptionally regulate tumor metabolism in prostate carcinogenesis, making them promising targets for therapeutic intervention.

## 4. Perspectives and Conclusions

Recent studies have revealed that long noncoding RNAs play a role in prostate cancer, serving as promising biomarkers for prostate cancer screening and risk stratification. And the well-studied prostate cancer-associated long noncoding RNAs are summarized in Table 1. Although the long noncoding RNAs we reviewed herein have demonstrated high specificity and sensitivity in detecting prostate cancer, their accuracy is not better than that of biopsy, even when multiple biomarkers are used. Moreover, polymorphisms in certain long noncoding RNAs are strongly correlated with risk prediction and stratification within or among various populations; nonetheless, relatively few individuals were examined in these previous studies. Therefore, performing such studies in a large cohort of individuals is necessary to further strengthen the promising evidence for long noncoding RNAs as biomarkers in prostate cancer risk prediction and stratification. Appreciating the critical role of long noncoding RNAs in prostate carcinogenesis and elucidating their mechanisms of action will help us to develop more specific drugs to interfere with malignant signaling and to specifically eradicate prostate cancer cells. Long noncoding RNAs (1) participate in prostate carcinogenesis through AR-mediated signaling pathways; (2) act as oncogenes and tumor suppressor genes in prostate carcinogenesis; and (3) mediate prostate carcinogenesis by interfering with DNA repair and tumor metabolism. Hence, based on the roles of long noncoding RNAs in prostate cancer, it will be interesting to develop intracellular interventions to interfere with malignant signaling networks. Furthermore, based on the mechanisms of action of long noncoding RNAs in prostate carcinogenesis and development, there are two main strategies, down-regulating long noncoding RNAs and inducing anaplerosis. The goal of down-regulating long noncoding RNAs involves re-activating or silencing the expression of long noncoding RNAs that are critical for prostate carcinogenesis and development, whereas replenishing long noncoding RNAs involves re-activating the function of TS lncRNAs and activating repressed endogenous tumor suppressor pathways. However, many challenges remain in the development of long noncoding RNAs as therapeutic targets for prostate cancer, including the poor stability of RNA molecules outside the cell and the lack of specific, secure and effective vectors for intracellular delivery. Certain solutions may be developed; enveloping RNA molecules in nanoparticles or conjugating them to cholesterol moieties improves RNA stability, and targeting these molecules specifically to cancer cells by linking tumor-specific ligands to the surfaces of nanoparticles has also been investigated. Although there is a promising future for long noncoding RNA as a potential target in prostate cancer, the malignant signaling network is complicated, and there is insufficient data to explain the function and regulation of the discovered long noncoding RNAs. Thus, it will be necessary to identify additional long noncoding RNAs that are involved in prostate carcinogenesis, to develop new effective methods for predicting their target genes and to investigate their functional motifs and molecular structures to fully elucidate their regulatory mechanisms. Together, these long noncoding RNAs provide new insights into the complicated malignant signaling networks and novel strategies for prostate cancer diagnosis and therapy.

**Table 1:** Summary of the well-studied prostate cancer-related long noncoding RNAs.

Long Noncoding RNA	Locus	Expression Level	Role	Description	Reference
*DD3^PCA3^*	Chr9q21.2	high	Urine/Plasma-based biomarker	(1) Biomarker for prostate cancer detection; (2) Polymorphism in its promoter region is related to the risk of prostate cancer in Chinese men.	[23,41]
*MALAT1*	Chr11q13.1	high		Biomarker for prostate cancer detection and progression.	[31,32]
*PCAT-1*	Chr8q24.21	high		Associated with the risk of and susceptibility to prostate cancer.	[69]
*PCAT-18*	Chr18q11.2	high		Biomarker for prostate cancer metastasis.	[33]
*SChLAP1*	Chr2q31.3	high	Tissue-based biomarker	Biomarker for clinical outcome of prostate cancer.	[35]
*PlncRNA-1*	Chr21	high	AR-related modulator	Promotes prostate carcinogenesis through reciprocal communication with AR.	[49]
*CTBP1-AS*	Chr4p16.3	high		Promotes prostate cancer growth by (1) repressing CTBP1 via the recruitment of PSF and HDAC and (2) promoting cell cycle progression by inhibiting tumor suppressor genes via AR.	[51,52]
*Linc00963*	Chr9	high (especially in hormone-insensitive prostate cancer)		Participates in the transition from androgen-dependence to androgen-independence in the progression of prostate cancer via interaction with EGFP.	[58]
*PCAT-1*	Chr8q24.21	high	Oncogene	(1) Promotes prostate cancer proliferation and progression; (2) Regulates the cellular response to genotoxins by repressing the expression of BRCA2, causing a deficiency in homologous recombination.	[59]
*MALAT1*	Chr11q13.1	high		Maintains the tumorigenicity and progression of prostate cancer.	[60,61]
*SChLAP1*	Chr2q31.3	high (especially in aggressive prostate cancer)		Drives malignancy by antagonizing the SWI/SNF complex.	[62]
*ANRIL*	Chr9p21.3	high		An inhibitor of a tumor suppressor gene. (1) Impacts the ability of CBX7 to repress the function of INK4b/ARF/INK4a and cell senescence control; (2) Inactivates p15(INK4B) by binding to SUZ12.	[63,64]
*H19*	Chr11p11.5	low	Tumor suppressor gene	Represses prostate cancer metastasis through TGFβ1 via H19/miR-675.	[65–67]
*GAS5*	Chr1q25	low		Over-expression can reduce drug/UV resistance and promote apoptosis.	[68]
*PRNCR1*	Chr8q24.21	high		Maintains prostate cancer susceptibility.	[53,56,57]
*PCGEM1*	Chr2q32	high		(1) Promotes proliferation and colony formation and inhibits the apoptosis of prostate cancer cells; (2) Promotes prostate cancer growth predominantly by regulating tumor metabolism via c-myc; (3) Polymorphisms are associated with the risk of prostate cancer in Chinese men.	[38,40,53,54,57,70]
